# Construction and validation of a multi-function artificial intelligence–assisted system for pressure injury recognition

**DOI:** 10.3389/fphys.2026.1773031

**Published:** 2026-02-18

**Authors:** Zhenni Wang, Yueping Xu, Kaijian Xia, Yiqi Dai, Xiaodan Xu, Jian Chen

**Affiliations:** 1 Gastroenterology Department, Changshu Hospital Affiliated to Soochow University, Suzhou, China; 2 Nursing Department, Changshu Hospital Affiliated to Soochow University, Suzhou, China; 3 Key Laboratory of Medical Artificial Intelligence and Big Data, Changshu Hospital Affiliated to Soochow University, Suzhou, China

**Keywords:** automated staging, deep learning, pressure injury, size measurement, YOLO

## Abstract

**Background:**

With the acceleration of population aging, the incidence of pressure injury (PI) continues to rise, making early identification and accurate staging essential for preventing disease progression and improving prognosis. Conventional manual assessment relies heavily on clinical experience and subjective judgment, limiting real-time, objective, and quantitative evaluation.

**Objective:**

This study aimed to develop and validate an artificial intelligence model based on the YOLOv11 neural network that integrates automatic PI detection, intelligent staging, and wound size measurement, thereby enhancing the timeliness, accuracy, and objectivity of PI assessment.

**Methods:**

A total of 1,815 PI images collected from the electronic PI management systems of two medical centers between January 2021 and June 2025 were included. According to the 2019 National Pressure Ulcer Advisory Panel (NPUAP) guidelines, images were classified into six categories: Stage I, Stage II, Stage III, Stage IV, unstageable, and deep tissue injury. Transfer learning was applied to train YOLOv11 models of different scales (v11n/s/m/l/x). Lesion localization and staging performance were compared to identify the optimal model. Automatic wound size measurement was achieved by integrating ArUco marker recognition with pixel-to-centimeter conversion.

**Results:**

For bounding box localization, the YOLOv11s model demonstrated superior performance, with a precision of 0.854, recall of 0.766, mAP50 of 0.842, mAP^50–95^ of 0.629, and an inference speed of 4.8 ms per image. On the test set, overall staging classification accuracy reached 92.64%, with a sensitivity of 79.79%, specificity of 95.56%, and a false-positive rate of 4.44%. The highest accuracy was observed for deep tissue injury (96.45%), while Stage III showed the lowest accuracy (85.04%). In wound size measurement, PI-3DAS demonstrated high agreement with the reference standard, with a length mean absolute error (MAE) of 0.155 cm and intraclass correlation coefficient (ICC) of 0.996, and a width MAE of 0.137 cm and ICC of 0.994. The mean time for AI-based measurement was 0.691 s, representing a 36.8-fold reduction compared with manual measurement (25.414 s; P < 0.001).

**Conclusion:**

The YOLOv11-based PI-3DAS system enables automated PI detection, staging, and non-contact wound size quantification with high accuracy and consistency, while substantially improving measurement efficiency. This system provides a portable and practical tool to support clinical nursing assessment, therapeutic follow-up, and remote PI management.

## 1 Introduction

Pressure injury (PI), also referred to as pressure ulcer or decubitus ulcer, is defined as localized damage to the skin and/or underlying soft tissue caused by sustained pressure or pressure combined with shear forces, and may also be associated with medical devices or other external objects. Once PI develops, patients may suffer from pain, discomfort, and reduced mobility; in severe cases, it can result in serious infection, prolonged hospitalization, or even death, posing substantial challenges to both patients and healthcare systems (Me et al., 2019). The severity of PI ranges from non-blanchable erythema of intact skin to complete loss of skin and underlying tissues with exposed bone (Headlam and Illsley, 2005). The prevention and management of PI represent a central yet challenging aspect of clinical nursing practice. In China, tertiary hospitals have widely implemented dedicated PI training programs and established specialized nursing teams. Nevertheless, nurses with limited clinical experience may demonstrate reduced accuracy in PI staging due to insufficient professional knowledge, assessment skills, and systematic training. In clinical evaluation, beyond staging, wound size measurement is a crucial indicator for monitoring disease progression and therapeutic effectiveness. Currently, most healthcare institutions rely on manual measurement using rulers or flexible tapes, a process that is labor-intensive and contact-dependent, increasing the risk of cross-infection. Moreover, such measurements are highly susceptible to variations in imaging angle, lighting conditions, and examiner subjectivity, resulting in limited reproducibility and objectivity (Haavisto et al., 2022; Boyko et al., 2018).

In recent years, driven by the rapid advancement of big data and cloud computing technologies, artificial intelligence (AI) has been increasingly adopted in the medical field, rendering computer-aided diagnosis a focal area of clinical research (Agnes et al., 2019). Šín et al. (2022) developed a machine learning model based on clinical data to predict the risk of PI occurrence, providing a structured risk assessment tool to support nursing decision-making. In the domain of medical image intelligence, researchers have further explored deep learning approaches for the automated detection and staging of PI. Aldughayfiq et al. (2023) proposed a YOLOv5-based model for automatic PI detection and classification into Stages I–IV, achieving an overall mAP of 76.9% on a large annotated dataset and outperforming conventional PI recognition methods. With continuous iterations of object detection algorithms, recent studies have demonstrated that optimized YOLOv8s models (mAP ≈84.2%, recall 82.3%) further enhance recognition accuracy for visually ambiguous stages such as Stage II, as well as for deep tissue injury and unstageable categories (Tusar et al., 2025). Despite these advances, most existing studies have focused on single tasks such as PI risk prediction, detection, or staging, and there remains a lack of integrated models that simultaneously address lesion detection, staging, and wound size measurement, thereby limiting standardized and objective quantification. Moreover, the majority of prior work has not incorporated explainable analysis, nor has it provided bedside tools suitable for real-time clinical application.

**FIGURE 1:**
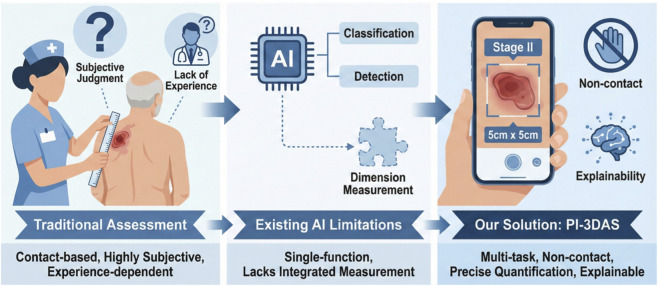
Schematic overview of the clinical application of the PI multi-function recognition system.

Accordingly, this study aims to develop a multi-function intelligent PI detection system that integrates automatic lesion detection, intelligent staging, and wound size measurement (Figure 1). A non-contact digital measurement approach is introduced, and Grad-CAM is employed to provide visual interpretability of the model’s decision-making process. In addition, the model is implemented as an interactive bedside tool to enhance the accuracy, objectivity, and clinical usability of PI assessment.

## 2 Materials and methods

### 2.1 Datasets

**FIGURE 2:**
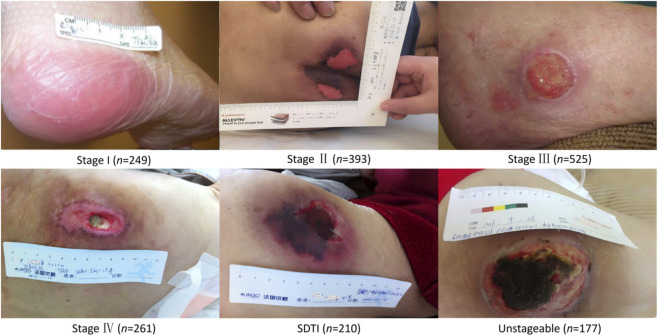
Representative images and distribution of quantities in the dataset.

**FIGURE 3:**
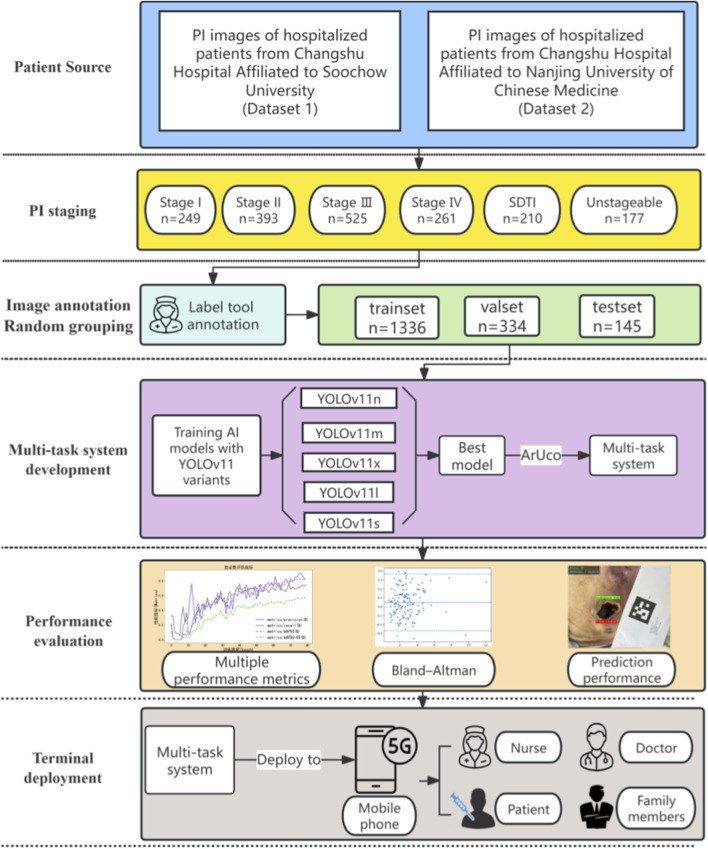
Research flowchart.

This study collected a total of 1,815 images of pressure injury (PI) from hospitalized patients between January 2021 and June 2025 at Changshu Hospital Affiliated to Soochow University (Dataset 1) and Changshu Hospital Affiliated to Nanjing University of Chinese Medicine (Dataset 2). All images were stored in a dedicated electronic PI management system. According to the 2019 guidelines for the prevention and treatment of pressure injury issued by the National Pressure Ulcer Advisory Panel (NPUAP) (Kottner et al., 2019), PIs were classified into six stages: Stage I (249 images), Stage II (393 images), Stage III (525 images), Stage IV (261 images), suspected deep tissue injury (SDTI; 210 images), and unstageable (177 images). Representative images for each stage are presented in Figure 2, and the overall study workflow is illustrated in Figure 3.

To prevent data leakage, patient-level separation was implemented during dataset splitting. All images from the same patient were assigned to the same data subset (training, validation, or test set), ensuring that the validation and test sets contained no images from patients in the training set.

### 2.2 Image acquisition and annotation

PI images were captured by inpatient nursing teams across different clinical departments during routine care and subsequently uploaded to the electronic pressure injury management system. Once a PI was identified, nurses used either medical handheld devices or personal smartphones to take photographs, ensuring that the camera-to-lesion distance was maintained between 40 and 65 cm. During image acquisition, careful handling was emphasized to minimize motion artifacts and ensure high image quality.

**FIGURE 4:**
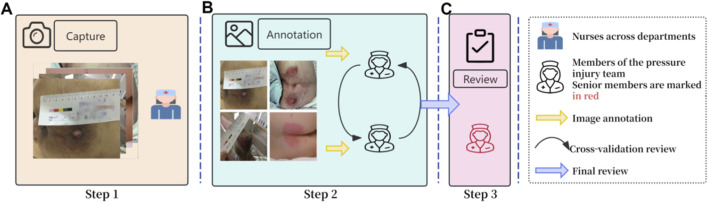
Image Annotation Process. **(A)** represents the first stage, in which clinical nurses from various hospital departments identify and photograph PI lesions; **(B)** denotes the second stage, where two members of the pressure injury specialist team independently annotate PI images from all six stages using rectangular bounding boxes and perform cross-validation; **(C)** indicates the third stage, in which a senior pressure injury specialist holding international wound ostomy certification reviews all annotations and makes the final adjudication.

**FIGURE 5:**
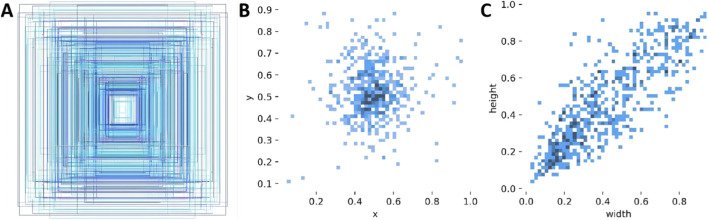
Image annotation display. **(A)** presents an aggregated overview of all annotated bounding boxes. **(B)** and **(C)** illustrate the distributions of bounding box center coordinates and width–height dimensions, respectively, where darker colors indicate higher frequencies.

The image annotation process was divided into three sequential stages, with participating nurses organized into three teams, each responsible for a specific stage. The detailed annotation workflow is illustrated in Figure 4. Only images that had undergone standardized annotation and verification were included in the training of the deep learning models. Rectangular bounding-box annotations were performed for all six PI stages using the LabelMe tool (v5.3.1). To ensure compatibility with model training, the LabelMe-generated JSON files were converted into YOLO format. Detailed examples of the bounding-box annotations are presented in Figure 5.

### 2.3 Deep learning network

#### 2.3.1 Image preprocessing

This study aimed to enhance the accuracy of AI-based PI image recognition through a series of image preprocessing and augmentation strategies. During training, online data augmentation techniques were employed (Athalye and Arnaout, 2023; Kang et al., 2019), whereby image data were dynamically modified in real time without generating additional image files. This approach ensured that the model was exposed to slightly varied images at each iteration, thereby improving its robustness to real-world image variability. Preprocessing steps included resizing images to 640 pixels while preserving the original aspect ratio, as well as applying random horizontal flipping with a probability of 50% to increase data diversity. In addition, RandomResize and RandomCrop techniques were used to introduce stochastic variations in image scale and spatial cropping, enhancing the model’s ability to recognize size variations and local features. Furthermore, HSVRandomAug provided by YOLOX (Qiu et al., 2023) was applied to randomly adjust the HSV color space, strengthening the model’s adaptability to variations in illumination and color conditions.

#### 2.3.2 Model training configuration

This study adopted a transfer learning strategy (Shin et al., 2016) and utilized five variants of the YOLOv11 model pretrained on the Common Objects in Context (COCO) dataset (Jocher et al., 2023), namely, nano (n), small (s), medium (m), large (l), and extra-large (x), representing increasing levels of model size and complexity. Model weights were initialized and subsequently fine-tuned by retraining all layers on the PI image dataset. During training, the optimizer was automatically selected, and the learning rate was adaptively adjusted according to the configuration file to optimize performance. The maximum number of training epochs was set to 100, with a batch size of 16 and a maximum of 30 detectable objects per image. Automatic mixed-precision training on the graphics processing unit (GPU) was enabled to improve computational efficiency. All training settings, including resizing images to 640 pixels, were strictly implemented according to the predefined configuration parameters. To mitigate overfitting, an early stopping mechanism was applied with a patience of 10 epochs, such that training was terminated if no improvement in validation performance was observed over 10 consecutive epochs.

#### 2.3.3 Multi-function system development

**FIGURE 6:**
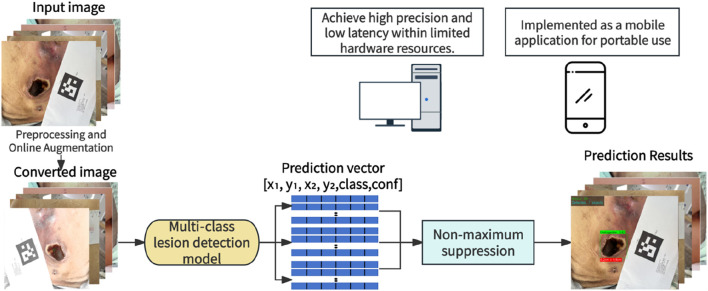
A multi-function system for PI automatic detection, staging, and dimensional measurement.

After identifying the optimal YOLOv11 model, the present study further extended it into a multi-function system integrating automatic lesion detection, intelligent staging, and physical size measurement (Figure 6). This three-in-one framework was designated PI-3DAS (Pressure Injury 3-Task Detection, Assessment, and Sizing), highlighting its unified design for simultaneously accomplishing lesion localization, staging, and dimensional quantification.

To enable objective size measurement, the system first establishes a conversion relationship between image pixels and real-world physical distances using Augmented Reality University of Cordoba (ArUco) markers. A standardized 5 cm × 5 cm ArUco marker (perimeter 20 cm) was generated using a custom script (available at: https://share.weiyun.com/sZ48DSxB) and placed on the same plane as the lesion during image acquisition. The OpenCV ArUcoDetector was then employed to automatically identify the four corner points of the marker and compute its pixel perimeter ( $P_{\text{pixel}}$ ). The pixel-to-centimeter conversion ratio was subsequently calculated using the formula: $pixel\_cm\_ratio=\frac{P_{pixel}}{P_{real}}$ , where the true perimeter P_real equals 20 cm. This ratio represents the number of pixels corresponding to 1 cm in the image and serves as the key parameter for dimensional conversion.

Following scale calibration, the input image is processed by the optimized YOLOv11 model. The model outputs include the lesion bounding box coordinates (x_1_, y_1_, x_2_, y_2_), the predicted staging category, and the associated confidence score. The pixel-based width ( $W_{pixel}$ ) and height ( $H_{pixel}$ ) of the lesion are obtained from the horizontal and vertical differences of the bounding box. Using the established conversion ratio, pixel dimensions are transformed into real physical distances, enabling automatic measurement of lesion length and width. The lesion length and width were calculated according to Equations 1, 2. According to the following formulas:

$$W_{cm}=\frac{W_{pixel}}{pixel\_cm\_ratio}$$ (1)

$$H_{cm}=\frac{H_{pixel}}{pixel\_cm\_ratio}$$ (2)

### 2.4 Model performance evaluation

Multiple evaluation metrics were employed to comprehensively assess the performance of the AI models. For deep learning–based object detection, evaluation focused on two key aspects: the accuracy of bounding box localization and the correctness of category prediction. Bounding box precision was quantified using mAP^50^ and mAP^50–95^, where mAP^50^ represents the mean average precision at an intersection-over-union (IoU) threshold of 0.5, and mAP^50–95^ denotes the mean average precision averaged across IoU thresholds ranging from 0.50 to 0.95 in increments of 0.05. To evaluate the model’s performance in lesion classification, sensitivity, specificity, accuracy, and false-positive rate were calculated. In addition, real-time processing capability was assessed using frames per second (FPS), which directly reflects the model’s inference speed and responsiveness in practical clinical scenarios.

### 2.5 Web application development

To facilitate clinical translation of the PI intelligent assessment model, an interactive web-based application named “PI-3DAS” was developed using the Streamlit framework. The application was implemented in Python and integrates the optimally trained YOLOv11 model as its core inference engine. The front end provides an intuitive user interface, while the back end employs the OpenCV and PIL libraries for image processing. After users upload clinical images, the system automatically executes an integrated three-step workflow of detection, staging, and measurement: lesions are first precisely localized and staged, followed by calculation of the pixel-to-centimeter conversion through recognition of ArUco markers in the image, enabling automatic quantification of wound dimensions (length and width). The results are displayed in real time as visually enhanced images and structured data outputs. Owing to its lightweight design, ease of deployment, and cross-platform compatibility, the application is well suited for practical clinical use.

### 2.6 Experimental platform

The computational platform used in this study was configured with an NVIDIA RTX A4000 GPU (16.9 GB of VRAM), an Intel Xeon E5-2680 v4 six-core processor, 30.1 GB of system memory, and 451.0 GB of storage. Development, training, and image processing of the deep learning models were conducted using PyTorch 1.10.1 with CUDA 11.3 support. The model development environment was based on Ultralytics YOLOv11.3.47, and the web application was implemented using Streamlit 1.36.0, all running under Python 3.9.

### 2.7 Statistical analysis

Data processing and analysis were performed using Python 3.9 and associated libraries, including Pandas 1.3.4 and NumPy 1.21.4. Continuous variables are presented as mean ± standard deviation or median (interquartile range), as appropriate, while categorical variables are expressed as frequencies and percentages. Model performance was evaluated using precision, recall, mean average precision (mAP), F1 score, sensitivity, specificity, and accuracy. For PI size measurement, accuracy was quantified using mean absolute error (MAE), root mean square error (RMSE), and mean absolute percentage error (MAPE). Agreement between AI-based and reference measurements was assessed using the intraclass correlation coefficient [ICC(A,1)], with values > 0.75 indicating good agreement, and Bland–Altman analysis was applied to evaluate systematic bias and the 95% limits of agreement (LoA). Differences in measurement time between AI-based and manual methods were compared using the Wilcoxon signed-rank test (nonparametric). A two-sided P value < 0.05 was considered statistically significant.

## 3 Results

### 3.1 Model training

**FIGURE 7:**
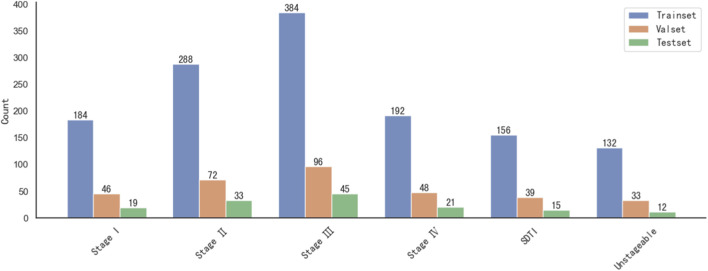
Distribution of images for each stage of PI.

A total of 1,815 PI images across different stages were included in this study and divided into a training set (1,336 images), a validation set (334 images), and a test set (145 images). The distribution of images by stage was as follows: Stage I, 249 images; Stage II, 393 images; Stage III, 525 images; Stage IV, 261 images; suspected deep tissue injury (SDTI), 210 images; and unstageable, 177 images. The detailed distribution is illustrated in Figure 7.

**FIGURE 8:**
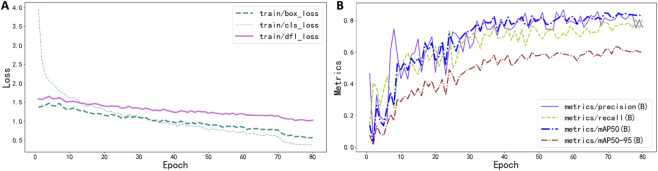
Changes in the loss function and performance metrics during YOLOv11 model training. **(A)** shows the variation of the loss function, while **(B)** illustrates changes in bounding box localization performance metrics. mAP50 denotes the mean average precision at an intersection-over-union (IoU) threshold of 0.5, and mAP^50–95^ represents the mean average precision averaged over IoU thresholds ranging from 0.50 to 0.95 in increments of 0.05.

During training, the loss function of the YOLOv11n model exhibited a clear downward trend, progressively decreasing and eventually stabilizing as the number of training epochs increased, indicating convergence toward an optimal solution (Figure 8A). Concurrently, the bounding box localization performance metrics showed an upward trajectory throughout training, with a rapid initial improvement followed by stabilization. Specifically, the model achieved a bounding box precision of 0.837 and a recall of 0.711. In addition, the mAP^50^ and mAP^50–95^ reached 0.808 and 0.558, respectively, demonstrating the robust object detection capability of the YOLOv11n model (Figure 8B).

### 3.2 Bounding box localization performance of different YOLO model variants

**FIGURE 9:**
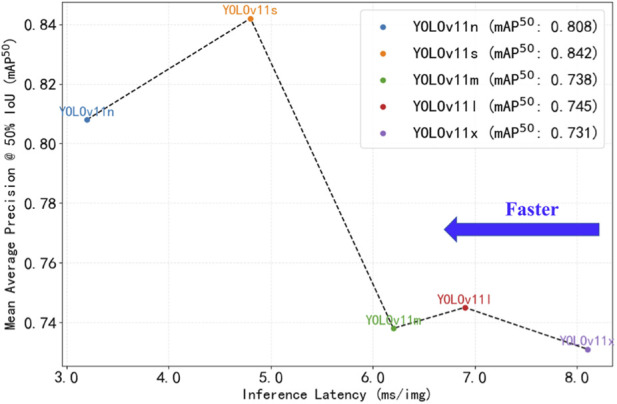
Performance of different versions of the YOLOv11 model.

The comparative performance of different models on the validation set is summarized in Table 1. To provide a comprehensive baseline comparison, the established YOLOv8s model was also evaluated under identical experimental conditions. Overall, YOLOv11s demonstrated the best bounding box localization performance, achieving higher precision (0.854), recall (0.766), mAP^50^ (0.842), and mAP^50–95^ (0.629) than the other variants. Compared with YOLOv8s, YOLOv11s showed improvements of 4.0% in precision, 5.8% in recall, 4.5% in mAP^50^, and 10.2% in mAP^50-95^, while maintaining comparable inference speed (4.8 ms vs. 4.5 ms per image). YOLOv11m and YOLOv11l showed slightly lower mAP^50^ and mAP^50–95^ values compared with YOLOv11s. In terms of inference speed, YOLOv11n exhibited the shortest inference time (3.2 ms per image), making it the fastest model; however, its mAP^50–95^ (0.558) and precision (0.837) were inferior to those of YOLOv11s. Conversely, YOLOv11x required the longest inference time (8.1 ms per image) and did not demonstrate advantages in either precision (0.698) or mAP^50^ (0.731). Taking both detection accuracy and inference speed into account, YOLOv11s achieved the most favorable performance balance in this study. The precision–latency trade-off curves for all models are shown in Figure 9.

**TABLE 1:** Performance of bounding box localization across different YOLO model versions.

YOLO Version	Precision	Recall	mAP^50^	mAP^50-95^	Speed (ms/img)
YOLOv8s	0.821	0.724	0.806	0.571	4.5
YOLOv11n	0.837	0.711	0.808	0.558	3.2
YOLOv11s	0.854	0.766	0.842	0.629	4.8
YOLOv11m	0.767	0.621	0.738	0.522	6.2
YOLOv11l	0.794	0.642	0.745	0.532	6.9
YOLOv11x	0.698	0.715	0.731	0.496	8.1

mAP^50^ and mAP^50–95^ denote the mean average precision at IoU thresholds of 0.5 and 0.50–0.95 (step = 0.05), respectively. Speed indicates the average inference time per image (ms) on an NVIDIA RTX A4000 GPU.

### 3.3 Classification performance of the YOLOv11s model on the test set

With respect to classification accuracy across different PI stages, the YOLOv11s model achieved an overall accuracy of 92.64%, with a sensitivity of 79.79%, specificity of 95.56%, and a false-positive rate of 4.44% across all categories. Among the six PI stages, the highest classification accuracy was observed for SDTI at 96.45%, whereas Stage III exhibited the lowest accuracy at 85.04%. Detailed performance metrics for each stage are presented in Table 2.

**TABLE 2:** Classification performance of the YOLOv11s model on the test set (%).

Category	Accuracy (%)	Sensitivity (%)	Specificity (%)	Precision (%)	F1 Score (%)	FPR(%)
StageI	95.58	85.00	97.85	89.47	87.18	2.15
Stage II	89.26	75.86	93.48	78.57	77.19	6.52
Stage III	85.04	77.27	89.16	79.07	78.16	10.84
Stage IV	93.10	76.19	96.84	84.21	80.00	3.16
SDTI	96.45	91.67	97.00	78.57	84.62	3.00
Unstageable	96.43	72.73	99.01	88.89	80.00	0.99
Macro-averaged	92.64	79.79	95.56	83.13	81.19	4.44

The overall (macro-averaged) values represent the equally weighted mean of the performance metrics (e.g., accuracy, sensitivity) across all PI, stages; False Positive Rate (FPR).

### 3.4 Relationship between precision, recall, F1 score, and confidence in the AI model

**FIGURE 10:**
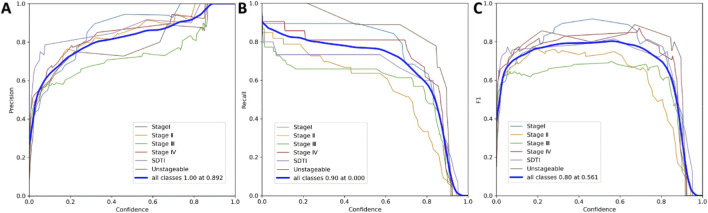
Relationship between precision, recall, F1 score, and confidence in the AI model. **(A)** shows the precision–confidence curve, **(B)** the recall–confidence curve, and **(C)** the F1 score–confidence curve.

The YOLOv11s object detection model was applied to localize PI lesions and estimate prediction confidence. During video analysis, the model performed continuous detection on each frame. The highest F1 score was achieved at a confidence threshold of 0.561 (Figure 10). At lower confidence levels, the model effectively balanced precision and recall, minimizing the risk of missing potential lesions during the initial screening phase—an aspect that is particularly critical in clinical settings, where final diagnosis relies on verification by specialized nursing teams. In typical object detection tasks, an appropriate trade-off between precision and recall is required, and the optimal cutoff point is generally determined at the peak F1 score. In real-world clinical applications, pressure injury specialist nurses review the model’s annotations; when the confidence threshold was ≤0.1, the F1 score increased rapidly and subsequently showed a gradual upward trend.

### 3.5 PI-3DAS performance in PI size measurement

The wound size measurement performance of PI-3DAS was evaluated on the test set (n = 145). Compared with the reference standard, PI-3DAS demonstrated small measurement errors for both length and width (Table 3): for length, MAE was 0.155 cm, RMSE was 0.211 cm, and MAPE was 3.68%; for width, MAE was 0.137 cm, RMSE was 0.168 cm, and MAPE was 5.85%. Under the absolute error threshold of |Δ| ≤ 0.5 cm, the success rates for length and width measurements were 96.6% and 100.0%, respectively; under the relative error threshold of ≤10%, the corresponding success rates were 93.1% and 82.8%.

**TABLE 3:** Accuracy and agreement of PI size measurements between PI-3DAS and the reference standard.

Measurement	MAE (cm)	RMSE (cm)	MAPE (%)	Success Rate (≤0.5 cm),%	Success Rate (Rel≤10%),%	ICC(A,1) [ICC 95% CI]	Bias (cm)	LoA (cm)
Length	0.155	0.211	3.68	96.6	93.1	0.996 (0.994–0.998)	0.044	−0.361 to 0.449
Width	0.137	0.168	5.85	100	82.8	0.994 (0.989–0.996)	0.047	−0.270 to 0.364

ICC(A,1), intraclass correlation coefficient (two-way random-effects, absolute agreement); LoA, limits of agreement.

**FIGURE 11:**
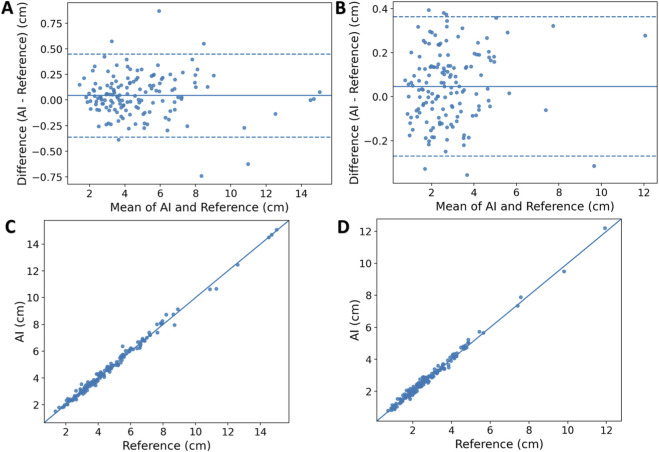
Agreement between PI-3DAS and the reference standard for PI size measurements. **(A,B)** show Bland–Altman plots for length and width, respectively, illustrating the mean difference (solid line) and the 95% limits of agreement (dashed lines). **(C,D)** present scatter plots comparing AI-derived measurements with the reference standard for length and width, respectively.

Agreement analysis indicated excellent concordance between PI-3DAS and the reference standard (Table 3; Figures 11A,B), with ICC(A,1) values of 0.996 (95% CI: 0.994–0.998) for length and 0.994 (95% CI: 0.989–0.996) for width. Bland–Altman analysis revealed biases close to zero with a slight positive bias (AI measurements marginally higher than the reference standard) (Figures 11C,D): length bias of +0.044 cm with limits of agreement (LoA) from −0.361 to 0.449 cm, and width bias of +0.047 cm with LoA from −0.270 to 0.364 cm.

In terms of efficiency, PI-3DAS markedly reduced the time required for single-case measurement. The median and mean times for AI-based measurement were 0.694 s and 0.691 s, respectively, compared with 23.662 s and 25.414 s for manual measurement, representing an approximately 36.8-fold reduction in measurement time. The difference was statistically significant according to the Wilcoxon signed-rank test (P < 0.001).

### 3.6 Terminal deployment and visual interpretability

**FIGURE 12:**
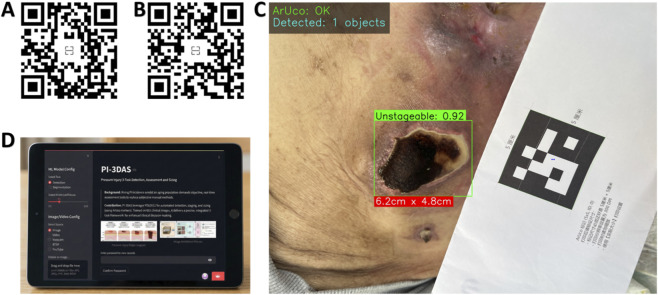
Terminal deployment and application examples of the PI-3DAS multi-function system. **(A)** QR code for mobile access to the web application; **(B)** application example in a video scenario with real-time PI detection, staging, and size measurement; **(C)** application example in an image scenario with automatic wound size measurement based on ArUco marker calibration; **(D)** user interface of the PI-3DAS web application supporting image/video upload and real-time inference.

Based on the best-performing YOLOv11s model, this study developed a multi-function AI-assisted system that integrates pressure injury (PI) lesion detection, automated staging, and wound size measurement, and deployed it as a web-based application using the Streamlit framework, designated PI-3DAS. To facilitate broad adoption across hospitals, nursing homes, and home-care settings—and to meet the needs of clinicians, nurses, patients, and students—users can access the system by scanning the QR code shown in Figure 12A or by visiting https://pi-3das-v2.streamlit.app/via a mobile browser. The user interface is illustrated in Figure 12D.

Figures 12B,C demonstrate representative applications of the system in video and image scenarios. Users may upload images or videos through the sidebar or capture images in real time using a camera on a mobile device or computer. Upon clicking the “Predict” button, the system automatically performs PI detection, staging, wound size measurement, and heatmap generation. The application is cross-platform, user-friendly, and shareable, enabling seamless use on both mobile and desktop devices.

**FIGURE 13:**
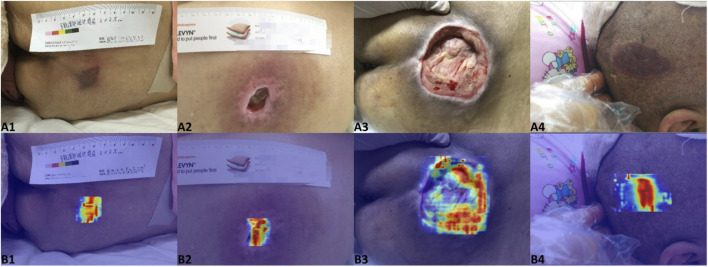
Grad-CAM visualizations of the AI model’s decision-making process. **(A1–A4)** show the original images, and **(B1–B4)** display the corresponding activation heatmaps overlaid on the original images.

To enhance model interpretability and support clinical decision-making, the system integrates Grad-CAM heatmap visualization (Figure 13). By selecting the “Visualize Heatmap” option in the sidebar, users can overlay class activation heatmaps onto the detection results, intuitively highlighting the lesion-specific regions that the model focuses on during diagnostic inference. This feature assists clinicians in validating the rationale and reliability of AI-based assessments.

## 4 Discussion

PI represents a major global health burden affecting millions of individuals worldwide, and its early detection and accurate staging are critical for preventing lesion progression and improving clinical outcomes. In Europe and North America, Stage II and above PIs are regarded as “never events.” Nevertheless, the incidence of PI remains alarmingly high, reaching up to 66% among patients undergoing prolonged surgical procedures and approximately 50% in intensive care units (Hajhosseini et al., 2020). In China, the accelerating aging population and uneven distribution of medical resources mean that specialized PI teams are predominantly concentrated in large tertiary hospitals, posing persistent challenges for early diagnosis and accurate staging in primary healthcare settings. Previous studies have reported PI healing rates ranging from only 5.1%–29.9% (Sun et al., 2017), which not only prolongs hospital stays but also substantially increases healthcare costs (Haesler et al., 2022). Against this background, the present study developed and validated PI-3DAS, a multi-function AI-assisted system that delivers a closed-loop output encompassing lesion detection, automated staging, and wound size measurement within a unified framework, while also achieving explainable visualization and terminal deployment.

Effective PI management depends critically on early and accurate identification. For less experienced healthcare professionals, precise PI staging remains challenging and may be compromised by insufficient training, limited information, observer bias, and the heterogeneous appearance of wounds (LeBlanc et al., 2019; Bates-Jensen et al., 2019). In addition, patient-related factors such as skin pigmentation, age, and overall health status can further confound visual assessment (Boyko et al., 2018). Consequently, there is an urgent need for more efficient, accurate, and objective automated methods for PI detection and staging. Computer-aided diagnosis has emerged as a focal area of clinical research. Šín et al. (2022) applied machine learning to clinical data from intensive care unit patients to predict PI risk, reporting an accuracy of 96.0% using a random forest model. In the field of PI image analysis, (Aldughayfiq et al., 2023) developed a YOLOv5-based model that achieved an mAP50 of 0.769. In contrast, by adopting the more advanced YOLOv11 architecture, our study achieved superior detection performance (mAP50 of 0.842) and demonstrated robust automated PI staging, with an overall accuracy of 92.64%, sensitivity of 79.79%, and specificity of 95.56%. Notably, the model achieved particularly high accuracy for suspected deep tissue injury, reaching 96.45%.

Beyond staging, objective quantification of wound size represents a critical challenge in PI management. Measurements of wound dimensions, including length, width, and area, directly influence staging decisions, treatment planning, and evaluation of healing progression.The NPUAP Pressure Ulcer Scale for Healing (PUSH) Tool 3.0, which is widely used in clinical practice, instructs clinicians to measure the greatest length (head to toe) and the greatest width (side to side) and multiply these measurements to estimate surface area. Although the length × width method may overestimate wound area by approximately 44% for irregularly shaped wounds (Sahbudak and Gunes, 2026), it remains the predominant measurement approach in clinical settings due to its simplicity, practicality, and acceptable reproducibility for monitoring wound status over time. The EPUAP/NPIAP/PPPIA 2019 International Clinical Practice Guideline continues to recommend standardized wound size measurement using this approach.

Conventional measurement methods rely primarily on rulers or flexible tapes, which are not only labor-intensive but also involve direct contact with damaged tissue, making it difficult to comply with aseptic and minimal-contact principles. Previous studies have suggested that digital wound measurement may serve as a non-contact and objective alternative (Liu et al., 2023). Liu et al. (2023), Aldughayfiq et al. (2023) combined U-Net and Mask R-CNN with a LiDAR camera to achieve PI region segmentation and size measurement; however, this approach depends on additional hardware and still yielded a mean relative error of 26.2%, limiting its applicability in routine ward and bedside settings. Aldughayfiq et al. (2023) developed a YOLOv5-based model for automated PI detection and staging, which improved recognition efficiency and staging consistency, but did not address automatic wound size quantification. In contrast, the present study integrates YOLOv11 with ArUco marker–based calibration to construct a unified multi-function system encompassing detection, staging, and size measurement, thereby enabling non-contact digital quantification. PI-3DAS demonstrated excellent agreement in both length and width measurements (ICC >0.99), with errors controlled within 0.2 cm, and achieved approximately a 37-fold improvement in measurement efficiency compared with manual methods. This approach effectively addresses the limitations of previous studies with respect to objective wound size quantification and practical bedside applicability.

Beyond performance validation, the clinical value of an AI system fundamentally depends on its deployability and credibility. In this study, the best-performing YOLOv11s model was deployed as an interactive web-based application (PI-3DAS) using the Streamlit framework. In contrast to most existing PI intelligent systems, which remain limited to algorithmic validation or rely on high-performance servers and specialized hardware, the proposed system achieves lightweight deployment without additional hardware requirements. Users can perform PI image or video analysis directly through mobile or desktop browsers, enabling flexible application across hospital wards, nursing homes, and home-care settings. To further enhance the transparency of model decision-making, Grad-CAM visualization was integrated into the system. The resulting heatmaps indicate that the model’s attention is primarily focused on clinically relevant regions, such as wound margins and areas of tissue abnormality, which are consistent with key clinical assessment criteria. This interpretability feature facilitates healthcare professionals’ understanding of AI-generated results and strengthens their confidence in AI-assisted evaluations.

This study has several limitations. At present, wound size measurement is primarily based on two-dimensional images and does not incorporate three-dimensional information such as wound depth. Additionally, the current bounding box-based measurement method aligns with routine clinical ruler measurements, providing length and width estimates rather than precise wound area. While pixel-wise segmentation approaches (e.g., U-Net) may offer more accurate area quantification, they require significantly greater annotation burden and computational complexity. Future studies may consider integrating segmentation-based methods for applications where precise area measurement is clinically indicated. Furthermore, although the dataset was derived from two independent medical centers, broader external validation across diverse healthcare settings and patient populations is needed to further establish the system’s generalizability.

## 5 Conclusion

In summary, this study developed and validated a multi-function AI-assisted system (PI-3DAS) based on the YOLOv11 neural network that integrates automated pressure injury detection, staging, and wound size measurement. The results demonstrate that the system achieves high accuracy and consistency in lesion recognition, automated staging, and wound dimension quantification. Moreover, through terminal deployment and explainable visualization, PI-3DAS enhances its usability and reliability in clinical nursing practice.

## Data Availability

The raw data supporting the conclusions of this article will be made available by the authors, without undue reservation.
